# *In vitro* evaluation of tigecycline synergy testing with nine antimicrobial agents against *Enterobacter cloacae* clinical strains

**DOI:** 10.3389/fmicb.2024.1490032

**Published:** 2024-10-18

**Authors:** Lukasz Korczak, Piotr Majewski, Krzysztof Rombel, Dominika Iwaniuk, Pawel Sacha, Mateusz Modzelewski, Elzbieta Tryniszewska

**Affiliations:** Department of Microbiological Diagnostics and Infectious Immunology, Medical University of Bialystok, Bialystok, Poland

**Keywords:** *Enterobacter cloacae*, E-test method, multidrug-resistant, synergy testing, tigecycline

## Abstract

*Enterobacterales* (especially carbapenem-resistant) are considered an urgent threat to public health. The available antibiotic therapy is limited due to the increase of multidrug-resistant (MDR) strains. Tigecycline, a minocycline derivative, has emerged as a potential key agent in the treatment of MDR isolates. The aim of the study was to evaluate the synergistic effect of tigecycline in combination with nine antimicrobial agents—ceftazidime/avibactam, colistin, ertapenem, gentamicin, imipenem, levofloxacin, meropenem/vaborbactam, polymyxin B, and rifampicin. Eighty clinical *Enterobacter cloacae* strains were obtained from patients of two University Hospitals in Bialystok, Poland. The E-test method was used to determine synergistic interactions. Among all combinations, synergy was reported in 61% of cases, addition in 32%, and indifference in 7%. The highest synergy rates were observed in tigecycline combinations with: ceftazidime/avibactam (60/80; 75%), imipenem (60/80; 75%), polymyxin B (55/80; 68.75%) and rifampicin (55/80; 68.75%), while the lowest synergy rate was noted in tigecycline-levofloxacin (26/80; 32.5%). The tigecycline-gentamicin showed the highest rate of indifference; antagonism, was not observed in any combination. In conclusion, tigecycline appears more suitable for use in combination therapy rather than as monotherapy and can be effectively paired with various antimicrobial agents against MDR *E*. *cloacae*. Further research will be necessary to confirm these results.

## Introduction

*Enterobacter cloacae* is a gram-negative pathogen causing nosocomial infections and outbreaks in hospital settings. Infections can affect immunocompromised patients, such as neonates and premature infants; patients with chronic diseases, such as diabetes mellitus; and patients who are undergoing immunosuppressive therapy, such as injured or burned patients. Therefore, extended hospitalization in intensive care units (ICU) (>14 days) can also be an important risk factor (Bazaid et al., 2022; Qureshi et al., 2011). Gram-negative bacteria such as *E*. *cloacae* can develop multiple mechanisms of antibiotic resistance, including efflux pumps, pump regulators, enzymatic inactivation [via *tet*(X) genes], or heteroresistance (Ruppé et al., 2015; Zhong et al., 2014; Stojowska-Swedrzyńska et al., 2021). The phenomenon of efflux pumps can lead to development of the MDR phenotype in bacterial strains (Li et al., 2019). Interestingly, the number of MDR strains is still increasing and is a major threat to public health (Catalano et al., 2022).

Tigecycline (a 9-t-butyl glycol amide derivative of minocycline) is becoming a key therapeutic option in the development of multidrug resistance. Multidrug resistance is defined as non-susceptibility to ≥1 agent in ≥3 antimicrobial categories (Cosentino et al., 2023). The mechanism of action is associated with inhibition of bacterial protein translation toward bacterial ribosome 30S subunit (Yaghoubi et al., 2022). Tigecycline exhibits a broad spectrum of antimicrobial action including gram-positive pathogens (*Staphylococcus aureus*, methicillin-resistant *S. aureus, Streptococcus pneumoniae, Enterococcus* spp., vancomycin-resistant strains), gram-negative microorganisms [*Enterobacterales*, extended-spectrum-beta-lactamase (ESBL) producers and carbapenem-resistant (CRE) strains], anaerobes, and atypical microorganisms (Yaghoubi et al., 2022). Tigecycline is used for the treatment of complicated abdominal infections, complicated skin infections, and community-acquired pneumonia as a monotherapy (Yaghoubi et al., 2022). Due to the fact that tigecycline can overcome major mechanisms of resistance, tigecycline-resistant strains are still developing (Goodarzi et al., 2021). Combination therapy may be a solution when resistance involves broad-spectrum antibiotics.

In the era of multidrug resistance, studying antimicrobial combinations *in vitro* is becoming increasingly important. Interestingly physicians prefer combination therapy over monotherapy, due to *in vitro* experiments showing synergistic effects of combinations of various antibiotics (Tumbarello et al., 2018). Moreover, combination therapy exhibited lower mortality rates vs. monotherapy, especially in bloodstream infections and carbapenemase-producing *Enterobacterales* (CPE) (Schmid et al., 2019). *Enterobacterales* is a large group of gram-negative organisms including *E. cloacae*. Commonly used antibiotic combinations against *Enterobacterales* include tigecycline with polymyxin, tigecycline with aminoglycoside, and tigecycline with rifampicin or fosfomycin (Papst et al., 2018).

*In vitro* synergy testing can be assessed with different methodologies: the checkerboard method, time-kill assays (TKA), the multiple-combination bactericidal test (MCBT), and the E-test method (Doern, 2014). The checkerboard method evaluates the effectiveness of antimicrobial combinations tested in serial 2-fold dilutions at clinically relevant concentrations, typically including antibiotics from different classes. Limitations of that method include the requirement for a large amount of reagents and resources to evaluate different combinations. Moreover, only two antimicrobials can be tested simultaneously; combinations of three or four antimicrobial agents are not possible (Saiman, 2007). MCBT is preferred to test combinations of more than two antibiotics. The concentration used in MCBT is determined by what can be assessed in the patient's serum. Unlike checkerboard synergy testing, MCBT only evaluates fixed concentrations (Aaron et al., 2000). TKAs use the principle of MCBT by assessing activity over 48 h instead of a single point. Unlike MCBT, TKA evaluates the rate of killing, providing more a relevant outcome for patients (Doern, 2014). E-test methods are based on the diffusion of a continuous concentration gradient of antibiotic from an impregnated strip on a solid agar. E-tests are placed on an agar medium inoculated with the tested microorganism and incubated overnight. Then minimum inhibitory concentration (MIC) is determined by the point, where the no-growth zone touches the strip. There are two modifications of the E-test method. In the first modification, two strips are placed perpendicularly, intersecting at the MIC for each antimicrobial when tested individually. The second modification involves placing the first strip with antibiotic on the agar inoculated with the tested microorganism. After 60 min, the strip is removed and the second strip is placed in the same position (Doern, 2014; Lewis et al., 2002).

The aim of the study was to assess the synergistic effect of tigecycline *in vitro* combined with colistin, ceftazidime/avibactam, ertapenem, gentamicin, imipenem, levofloxacin, meropenem/vaborbactam, polymyxin B, and rifampicin among clinical *E*. *cloacae* strains.

## Materials and methods

### Clinical strains, media preparation, and antimicrobial agents

Eighty non-duplicate *E*. *cloacae* strains collected from patients of two hospitals—University Clinical Hospital and University Children's Clinical Hospital in Bialystok, Poland, between 2012 and 2023 were analyzed in this study. The origins of the strains and their sequence types (ST) and types of clinical materials are collected in Table 1. All isolates were preserved at −80°C in Tryptone Soya Broth (TSB) with 30% glycerol until further research. For biochemical identification of isolates and initial determination of the MIC, the VITEK 2^®^ system (bioMérieux SSC, France) was used. Strains were cultured on previously prepared MacConkey agar (OXOID LTD., Basingstoke, UK), a selective medium to culture gram-negative microorganisms. Determination of MIC for each antimicrobial agent was done on Mueller-Hinton agar (OXOID LTD., Basingstoke, UK). All E-tests (ceftazidime/avibactam, colistin, ertapenem, gentamicin, imipenem, levofloxacin, meropenem/vaborbactam, polymyxin B, rifampicin, tigecycline) were purchased from Liofilchem (Liofilchem Inc., Waltham, MA, USA).

**Table 1:** The origin of strains examined in the study, their sequence types and types of clinical material from which the strains originated.

**Hospital ward**	**Number of isolates**	**% of isolates**
Intensive care unit	44	55.00%
Hematology	10	12.50%
Urology	7	8.80%
Cardiology	6	7.50%
Surgery	5	6.30%
Neurology	4	5.00%
Gastrology	4	5.00%
**Sequence type (ST)**		
ST-89	44	55.00%
ST-50	2	2.50%
ST-90	2	2.50%
Other sequence types	32	40.00%
**Type of material**		
Respiratory tract	30	37.50%
Urine	24	30.00%
Wounds/Pus	12	15.00%
Blood	8	10.00%
Catheter	4	5.00%
Gastrointestinal tract	2	2.50%

### Antimicrobial susceptibility testing (MIC determination)

The MIC of each antibiotic was assessed using the E-test method according to the manufacturer's instructions. To perform AST, the bacterial inoculum was prepared to a density of 0.5 on the McFarland scale measured by the turbidimetric method. Strains were then plated onto Mueller-Hinton agar and E-test strips were placed on the medium. Plates were incubated at 35°C for 18–20 h. Interpretation of AST was performed according to guidelines of the European Committee on Antimicrobial Susceptibility Testing (EUCAST 2024, version 14.0) (The European Society of Clinical Microbiology Infectious Diseases, 2024). In order to maintain consistency and ensure an appropriate methodology in our study, we decided to determine the MIC values for colistin and its combinations with tigecycline using E-test method, despite EUCAST and CLSI recommendations for using the microdilution method. This decision was made after reviewing the literature, which indicated a high level of agreement between the microdilution and E-test methods. The concordance between the two methods ranged from 86 to 98% (García-Meniño et al., 2020; Arroyo et al., 2005; Reese et al., 2018).

**Figure 1:**
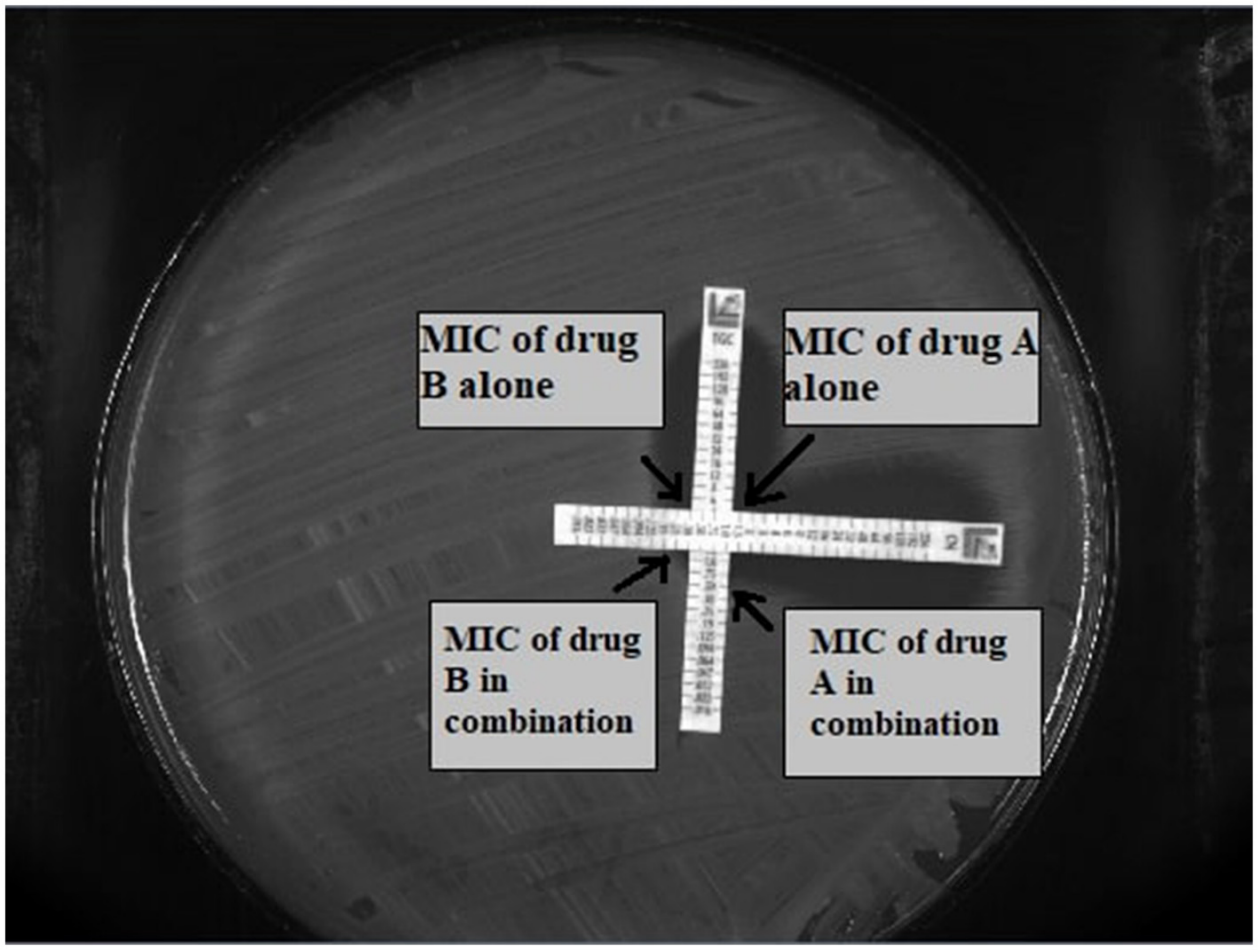
Principle of the E-test method. A—drug A, B—drug B.

AST was performed using the E-test MIC:MIC cross-formation method described by White et al. (1996). E-test strips were placed on the Mueller-Hinton agar in a cross formation, intersecting at a 90° angle at their MICs for the tested strain. Plates were incubated at 35–37°C for 18–20 h. After this time the zones of inhibition were read as described previously (Doern, 2014). The principle of method is illustrated in Figure 1. Moreover the fractional inhibitory concentration (FIC) index was calculated for each antibiotic in each combination. The formula to calculate the FIC index is as follows: *FIC INDEX* = (*MIC of drug A in combination*/*MIC of drug A alone*) + (*MIC of drug B in combination*/*MIC of drug B alone*).Synergy was defined as FIC index of ≤ 0.5; additive effect was defined as FIC index >0.5 to ≤ 1.0; indifference was defined as FIC index >1.0 to ≤ 4.0; and antagonism was defined as FIC index > 4.0 (Doern, 2014).

### Detection of AmpC β-lactamases and New Delhi metallo-β-lactamases

All tested strains were analyzed for the presence of AmpC β-lactamases and New Delhi metallo-β-lactamases. The presence of AmpC was confirmed using cloxacillin-supplemented agar with an evaluation of the differences in the inhibition zones between cefotaxime and imipenem discs. New Delhi metallo-β-lactamases were detected using O.K.N.V.I. RESIST-5 -*in vitro* rapid diagnostic tests for detection in bacterial culture. Tests were purchased from Coris BioConcept (Coris BioConcept, Gembloux, Belgium).

### Basic statistical tools

Basic statistical tools (such as median, mean, minimum, maximum and standard deviation) were used to calculate MIC and FIC values for antibiotics alone and in combination, as well as the median and mean FIC values to obtain a more complete picture of the results and a better understanding of its characteristics. STATA 17 (StataCorp, 2021) was used to assess these data.

## Results and discussion

Eighty isolates were examined *in vitro*, among which thirty-six (45%) were found to be susceptible to tigecycline. MICs for tigecycline ranged from 0.19 to 8 mg/L. Among the eighty isolates, forty-four of them were non susceptible to tigecycline (55%). For certain bacterial strains the MIC values exceeded the maximum value of the E-test strip. All examined MIC ranges for antimicrobial agents are presented in Table 2. During AST the phenomenon of heteroresistance was observed (subpopulation of bacterial cells exhibited a higher MIC value than the majority of the population). In that case higher MIC values were recorded. Sixty strains were chromosomal AmpC β-lactamases hyperproducers, and two of the tested isolates were New Delhi metallo-β-lactamase (NDM)-producing bacteria.

**Table 2:** The MIC ranges of all antimicrobial agents in monotherapy and in combination with tigecycline and their standard deviations.

**Antibiotic**	**MIC range in monotherapy (mg/L)**	**MIC in monotherapy (SD)**	**MIC range in combination with tigecycline (mg/L)**	**MIC in combination (SD)**
Ceftazidime/avibactam	0.032–256	40.40	0.016–4	0.54
Colistin	0.75–256	28.62	0.047–6	1.12
Ertapenem	0.032–32	4.73	0.016–4	0.85
Gentamicin	0.38–1024	228.60	0.064–1024	163.58
Imipenem	0.125–32	7.06	0.016–6	0.95
Levofloxacin	0.032–32	14.38	0.003–32	7.71
Meropenem/vaborbactam	0.032–24	2.75	0.016–0.25	0.11
Polymyxin B	1.0–1024	114.20	0.094–4.0	0.66
Rifampicin	6.0–256	27.70	0.19–8.0	0.37
Tigecycline	0.19–8	1.17	–	–

**Figure 2:**
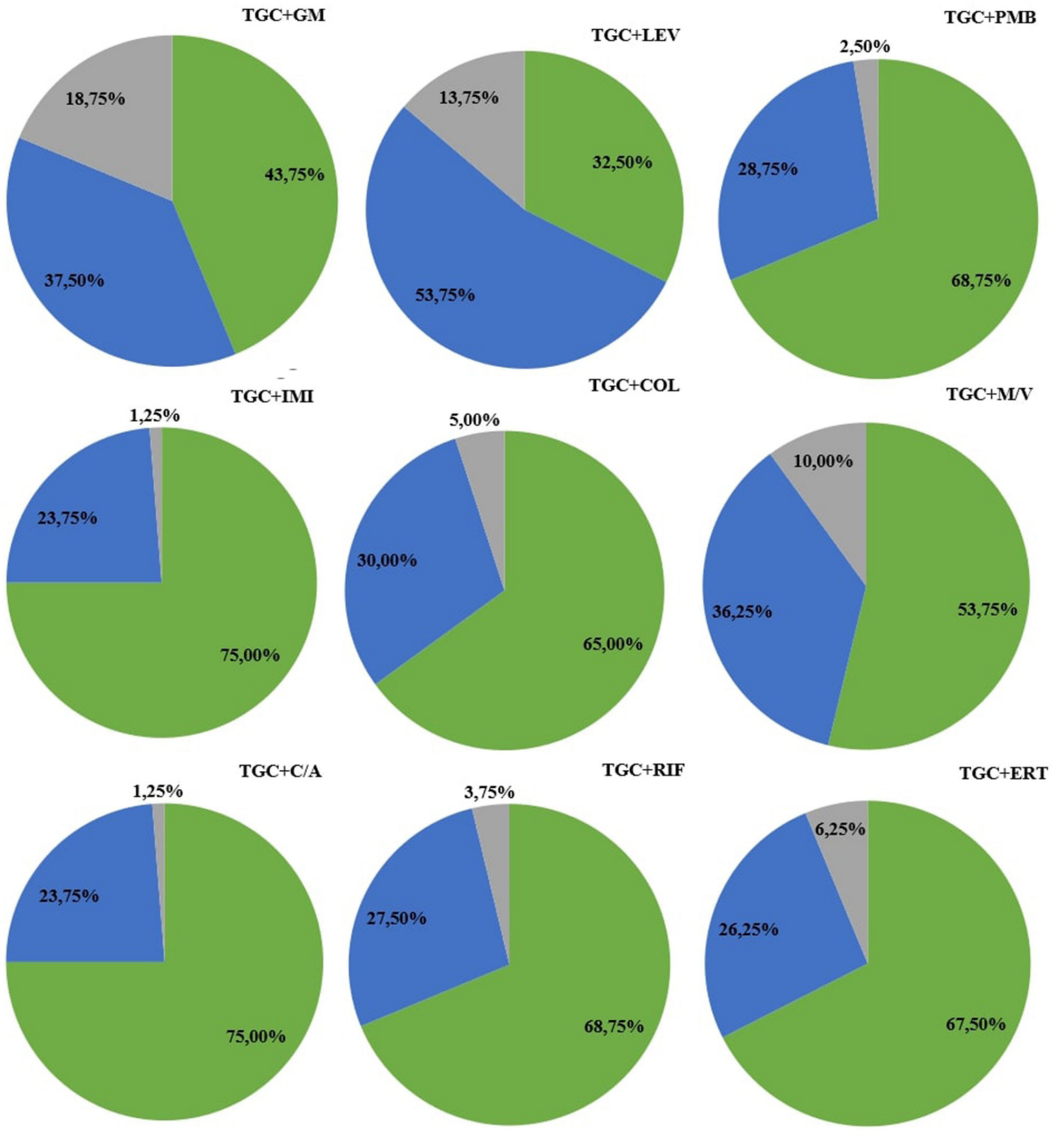
Percentage rates of antimicrobial synergy testing in combination with tigecycline. The green color represents synergy, blue indicates an additive effect, and gray represents indifference. Antimicrobial agents' abbreviations: C/A, ceftazidime/avibactam; COL, colistin; ERT, ertapenem; GM, gentamicin; IMI, imipenem; LEV, levofloxacin; M/V, meropenem/vaborbactam; PB, polymyxin B; RIF, rifampicin; TGC, tigecycline.

Among all nine combinations tested against eighty bacterial strains (total 720 samples for analysis) of antimicrobial agents used, synergy was observed in 440 samples (61%), addition in 230 cases (32%), and indifference in fifty cases (7%). Antagonism was not observed during research. The exact MIC values of antibiotics alone, MIC values in combination, and FICI values are presented in Supplementary Tables S1–S4. In tigecycline-ceftazidime/avibactam, 60/80 (75%) strains exhibited synergy. In tigecycline-colistin there were 52/80 (65%) synergistic strains. In tigecycline-ertapenem, 54/80 (67.5%) isolates exhibited synergy, while in tigecycline-gentamicin there were only 35/80 (43.75%) synergistic strains. Synergy was observed in 60/80 (75%) isolates in tigecycline-imipenem and 26/80 (32.50%) in tigecycline-levofloxacin. In tigecycline-meropenem/vaborbactam there were 43/80 (53.75%) synergistic strains, while tigecycline-polymyxin B exhibited 55/80 (68.75%) synergistic strains. In tigecycline-rifampicin there were 55/80 (68.75%) strains exhibiting synergy. Among nine combinations with tigecycline, the highest synergy rate was observed in tigecycline with imipenem (75%; 60/80) and tigecycline with ceftazidime/avibactam (75%; 60/80). The lowest synergy rate was observed in combination of tigecycline with levofloxacin (32.5%; 26/80). The highest rate of additive interaction was noted in tigecycline with levofloxacin (53.75%; 43/80), while the lowest was in tigecycline-imipenem and tigecycline-ceftazidime/avibactam (23.75%; 19/80). The highest indifference rate was observed in the tigecycline-gentamicin combination (18.75% 15/80), and the lowest rate in tigecycline-imipenem and tigecycline-ceftazidime/avibactam (1.25%; 1/80). All rates are presented in Figure 2. The most significant MIC reduction was noted in the levofloxacin combination; the tested strain's MIC was reduced from 32 mg/L in monotherapy to 0.064 mg/L in combination with tigecycline (500-fold reduction). Among all antibiotic combinations tested, there were fifteen cases where there was no reduction of MIC values in the combination compared to monotherapy. There were only two cases when MIC values in combination were higher compared to monotherapy. The most significant reduction in the average MIC value for tigecycline was observed in combination with ceftazidime/avibactam (7-fold reduction), whereas the poorest reduction was noted in tigecycline-gentamicin (3-fold reduction). The mean FIC index of all antibiotics tested was 0.5, while mean median was 0.44. A higher standard deviations in monotherapy indicates greater variability in the results. Lower standard deviations in combinations with tigecycline may suggest a more consistent and potentially more effective response to antibiotics than in monotherapy. All means, medians and standard deviations are presented in Table 3. Some of the resistant strains became susceptible during combination therapy with tigecycline. Among these, the highest amount of strains was observed in the tigecycline combinations with imipenem, meropenem/vaborbactam and polymyxin B (25/28, 11/11, 35/39, respectively). The lowest percentage of susceptible strains was observed in the combinations of gentamicin-tigecycline and levofloxacin-tigecycline (7/61, 6/59, respectively). All tested combinations and percentage rates are listed in Table 4.

**Table 3:** The FIC median, mean, standard deviation and MIC means with MIC reduction of all tested antimicrobial agents.

**Antibiotic**	**FIC index (median)**	**FIC index (mean)**	**FIC index (SD)**	**MIC antibiotic alone (mean) (mg/L)**	**MIC antibiotic in combination (mean) (mg/L)**	**MIC reduction**
Ceftazidime/avibactam	0.29	0.36	0.22	8.37	0.23	35.9-fold
Colistin	0.44	0.48	0.23	8.25	0.87	9.5-fold
Ertapenem	0.38	0.46	0.30	2.41	0.47	5.2
Gentamicin	0.58	0.69	0.41	174.24	63.32	2.8-fold
Imipenem	0.35	0.40	0.21	3.48	0.43	8.1-fold
Levofloxacin	0.63	0.73	0.45	20.34	7.39	2.8-fold
Meropenem/vaborbactam	0.47	0.53	0.32	0.61	0.03	22-fold
Polymyxin B	0.38	0.44	0.26	15.5	0.59	26-fold
Rifampicin	0.44	0.47	0.21	20.61	2.7	7.6-fold

**Table 4:** The synergy, addition, and indifference rates of isolated strains in all combinations with tigecycline.

**Antibiotic in combination with tigecycline**	**Synergy**	**Addition**	**Indifference**
Ceftazidime/ avibactam	60/80 (75.00%)	19/80 (23.75%)	1/80 (1.25%)
Colistin	52/80 (65.00%)	24/80 (30.00%)	4/80 (5.00%)
Ertapenem	54/80 (67.50%)	21/80 (26.25%)	5/80 (6.25%)
Gentamicin	35/80 (43.75%)	30/80 (37.50%)	15/80 (18.75%)
Imipenem	60/80 (75.00%)	19/80 (23.75%)	1/80 (1.25%)
Levofloxacin	26/80 (32.50%)	43/80 (53.75%)	11/80 (13.75%)
Meropenem/ vaborbactam	43/80 (53.75%)	29/80 (36.25%)	8/80 (10.00%)
Polymyxin B	55/80 (68.75%)	23/80 (28.75%)	2/80 (2.50%)
Rifampicin	55/80 (68.75%)	22/80 (27.50%)	3/80 (3.75%)

In the edge of post-antibiotic era combination therapy is becoming increasingly important. The TKA is regarded as the reference method for antibiotic synergy testing; however, it is time challenging and, therefore, it is not routinely used in microbiological laboratories. Interestingly, the E-test method appears to be more straightforward to perform and more cost efficient (Doern, 2014). Despite the simplicity of the E-test method, it is challenging to definitively determine which method is superior. Lewis et al. concluded that the E-test method was more accurate than the checkerboard method when comparing synergy methods in *Enterobacterales*. Surprisingly Papoutsaki et al. (2020) demonstrated poor correlation among all three methods (checkerboard, TKA, and E-test) particularly with tigecycline combinations in *Klebsiella pneumoniae* isolates (White et al., 1996). However, many studies reported a good agreement between E-test and checkerboard methods (Aktas, 2020; Orhan et al., 2005) or E-test with other synergy testing methods (White et al., 1996; Bonapace et al., 2000). White et al. conducted a comparative analysis of the three synergy testing methods to examine an efficacy of four antibiotic combinations against *Enterobacterales* family members (*P. aeruginosa, E. coli, E. cloacae*) and *S. aureus*. The study reported an agreement of 63–75% between the time-kill and E-test methods. Moreover, a 75% agreement rate was observed between checkerboard and E-test methods (White et al., 1996). Commonly used antibiotic combinations against *Enterobacterales* include tigecycline with polymyxin, tigecycline with aminoglycoside, and tigecycline with rifampicin or fosfomycin (Papst et al., 2018).

*In vitro* synergy testing can be assessed with different methodologies: the checkerboard method, time-kill assays (TKA), the multiple-combination bactericidal test (MCBT), and the E-test method (Doern, 2014). The checkerboard method evaluates the effectiveness of antimicrobial combinations tested in serial 2-fold dilutions at clinically relevant concentrations, typically including antibiotics from different classes. Limitations of that method include the requirement for a large amount of reagents and resources to evaluate different combinations. Moreover, only two antimicrobials can be tested simultaneously; combinations of three or four antimicrobial agents are not possible (Saiman, 2007). MCBT is preferred to test combinations of more than two antibiotics. The concentration used in MCBT is determined by what can be assessed in the patient's serum. Unlike checkerboard synergy testing, MCBT only evaluates fixed concentrations (Aaron et al., 2000). TKAs use the principle of MCBT by assessing activity over 48 h instead of a single point. Unlike MCBT, TKA evaluates the rate of killing, providing more a relevant outcome for patients (Doern, 2014). E-test methods are based on the diffusion of a continuous concentration gradient of antibiotic from an impregnated strip on a solid agar. E-tests are placed on an agar medium inoculated with the tested microorganism and incubated overnight. Then minimum inhibitory concentration (MIC) is determined by the point, where the no-growth zone touches the strip. There are two modifications of the E-test method. In the first modification, two strips are placed perpendicularly, intersecting at the MIC for each antimicrobial when tested individually. During our research we decided to analyze various *E. cloacae* isolates, particularly MDR/XDR strains. The E-test method was employed in our research owing to its simplicity and the promising results it has demonstrated. In this study, the antibiotic synergy testing was performed for tigecycline with nine antimicrobial agents (ceftazidime/avibactam, colistin, ertapenem, gentamicin, imipenem, levofloxacin, meropenem/vaborbactam, polymyxin B, and rifampicin). The rate of tigecycline susceptibility in monotherapy was 45% (36/80 isolates). Interestingly the susceptibility rates in the previous studies ranged from 58 to 86% (Tang et al., 2019; Evren et al., 2013; Lutgring et al., 2020). However, Nulsopapon et al. (2021) demonstrated that only 20% of strains were susceptible to tigecycline. This may be related to prior antibiotic use or horizontal gene transfer (HGT) and the dissemination of tigecycline resistance genes (Korczak et al., 2024).

The highest synergy rate was observed in tigecycline-ceftazidime/avibactam and tigecycline-imipenem combinations (75% of strains). In prior studies synergy rates of tigecycline-ceftazidime/avibactam ranged from 3.0 to 12.5% among *Enterobacterales*. Moreover, indifference rates were significantly higher in prior studies (67–87.5%) in comparison to our results (1.25%) (Wang et al., 2021; Ojdana et al., 2019). The tigecycline-imipenem synergy ratios varied from 9 to 88% in previous studies. Indifference rates were significantly higher than reported in this study (50–91% vs. 1.5%) (Evren et al., 2013; Goel et al., 2021; Dundar et al., 2018). Tigecycline-polymyxin B (TGC-PMB) and tigecycline-rifampicin (TGC-RIF) synergy rates were also high (68.75%). Prior studies revealed synergy rates at 29–35% for TGC-PMB and 22.6–60% for TGC-RIF (Lim et al., 2011; Huang et al., 2024). As the data in Table 2 suggest, due to the highest MIC values of polymyxin B and rifampicin in monotherapy, a combination therapy should be considered in patients treated with these antimicrobial agents. Moreover, TGC-RIF combination could suppress the expressions of efflux pump genes, like *rarA* and *acrB*, and might inhibit development of resistance in carbapenem-resistant *Klebsiella pneumoniae* strains in comparison to monotherapy (Shi et al., 2023).

The lowest synergy ratios were 32.50 and 43.75% for tigecycline-levofloxacin (TGC-LEV) and tigecycline-gentamicin (TGC-GM), respectively. Prior studies' synergy rates range from 20 to 80% for TGC-LEV and 8.2% for TGC-GM (Nulsopapon et al., 2021; Petersen et al., 2006). The indifference rate in the TGC-GM combination was similar (22.4% in prior study vs. 18.75% in our study) (Nulsopapon et al., 2021; Zhang et al., 2019).

In our study the tigecycline-colistin (TGC-COL) combination exhibited the synergy rate of 65%, while addition and indifference rates were 30 and 5%, respectively. None of the strains displayed antagonism. Dundar et al. reported the synergy of TGC-COL in 36% of strains. Additionally 8% of isolates exhibited antagonism. The remaining strains (56%) were indifferent or additive (Dundar et al., 2018). Betts et al. (2014) reported 47% of synergy and 53% of indifference and addition. Interestingly they also reported that the TGC-COL combination was definitely more effective against *E. coli, E. cloacae*, and *K. pneumoniae* than monotherapy.

Carbapenem-resistant *Enterobacterales* (CRE) is recognized as a critical group in the WHO Bacterial Priority Pathogens List (World Health Organization, 2024). Currently, the therapeutic options for treatment of CRE strains are limited. Even the effectiveness of newer beta-lactamase inhibitors like meropenem/vaborbactam depends on the specific types of carbapenemases produced by CRE isolates, which may depend on the region. Moreover, combination therapy is favored over monotherapy for treatment of CRE infections (Gutiérrez-Gutiérrez et al., 2017; Ni et al., 2016). Tigecycline-meropenem/vaborbactam synergy needs to be further investigated. Our research showed 53.75, 36.25, and 10% for synergy, addition, and indifference rates, respectively. Zhang et al. (2019) reported the tigecycline-meropenem combination with 50, 40, and 10% for synergy, addition, and indifference rates, respectively. Our results seem to be comparable to this prior study. Tigecycline-ertapenem synergy rate appeared to be higher (67.5%) in comparison to other studies with other carbapenem combinations (20–60%) (Petersen et al., 2006; Entenza and Moreillon, 2009).

This study has limitations: this research was performed only in *in vitro* settings, with only one method to assess antimicrobial synergy, and we did not evaluate patients' outcomes. However tigecycline combinations with almost all of the tested antimicrobial agents yielded promising results with high synergy ratings in the treatment of resistant *E*. *cloacae* isolates. Finally, our results may vary as our study included a larger group of isolates, whereas previous studies included only a few strains. Further research is needed for the evaluation of safety and efficacy of tigecycline combinations and to determine whether the E-test method can be considered as the reference method.

## Data Availability

The original contributions presented in the study are included in the article/Supplementary material, further inquiries can be directed to the corresponding author.

## References

[B1] Aaron S. D. Ferris W. Henry D. A. Speert D. P. Macdonald N. E. (2000). Multiple combination bactericidal antibiotic testing for patients with cystic fibrosis infected with Burkholderia cepacia. Am. J. Respir. Crit. Care Med. 161, 1206–1212. 10.1164/ajrccm.161.4.9907147 10764313

[B2] Aktas G. (2020). Activity of vancomycin combined with linezolid against clinical vancomycin-resistant Enterococcus strains. Arch. Med. Sci. 19, 189–193. 10.5114/aoms.2020.96400 36817687 PMC9897105

[B3] Arroyo L. A. García-Curiel A. Pachón-Ibañez M. E. Llanos A. C. Ruiz M. Pachón J.. (2005). Reliability of the E-test method for detection of colistin resistance in clinical isolates of *Acinetobacter baumannii*. J. Clin. Microbiol. 43, 903–905. 10.1128/JCM.43.2.903-905.2005 15695701 PMC548043

[B4] Bazaid A. S. Punjabi A. A. Aldarhami A. Qanash H. Alsaif G. Gattan H.. (2022). Bacterial infections among patients with chronic diseases at a tertiary care hospital in Saudi Arabia. Microorganisms 10:1907. 10.3390/microorganisms10101907 36296184 PMC9609889

[B5] Betts J. W. Phee L. M. Hornsey M. Woodford N. Wareham D. W. (2014). In vitro and in vivo activities of tigecycline-colistin combination therapies against carbapenem-resistant Enterobacteriaceae. Antimicrob. Agents Chemother. 58, 3541–3546. 10.1128/AAC.02449-14 24687491 PMC4068506

[B6] Bonapace C. R. White R. L. Friedrich L. V. Bosso J. A. (2000). Evaluation of antibiotic synergy against *Acinetobacter baumanni*i: a comparison with Etest, time-kill, and checkerboard methods. Diagn. Microbiol. Infect. Dis. 38, 43–50. 10.1016/S0732-8893(00)00163-2 11025183

[B7] Catalano A. Iacopetta D. Ceramella J. Scumaci D. Giuzio F. Saturnino C.. (2022). Multidrug resistance (MDR): a widespread phenomenon in pharmacological therapies. Molecules 27:616. 10.3390/molecules27030616 35163878 PMC8839222

[B8] Cosentino F. Viale P. Giannella M. (2023). MDR/XDR/PDR or DTR? Which definition best fits the resistance profile of *Pseudomonas aeruginosa*? Curr. Opin. Infect. Dis. 36, 564–571. 10.1097/QCO.0000000000000966 37930070 PMC10836784

[B9] Doern C. D. (2014). When does 2 plus 2 equal 5? A review of antimicrobial synergy testing. J. Clin. Microbiol. 52, 4124–4128. 10.1128/JCM.01121-14 24920779 PMC4313275

[B10] Dundar D. Duymaz Z. Genc S. Er D. K. Irvem A. Kandemir N.. (2018). In-vitro activities of imipenem-colistin, imipenem-tigecycline, and tigecycline-colistin combinations against carbapenem-resistant Enterobacteriaceae. J. Chemother. 30, 342–347. 10.1080/1120009X.2018.1516270 30663555

[B11] Entenza J. M. Moreillon P. (2009). Tigecycline in combination with other antimicrobials: a review of *in vitro*, animal and case report studies. Int. J. Antimicrob. Agents 34:8.e1–9. 10.1016/j.ijantimicag.2008.11.006 19162449

[B12] Evren E. Azap O. K. Çolakoglu S. Arslan H. (2013). *In vitro* activity of fosfomycin in combination with imipenem, meropenem, colistin and tigecycline against OXA 48-positive *Klebsiella pneumoniae* strains. Diagn. Microbiol. Infect. Dis. 76, 335–338. 10.1016/j.diagmicrobio.2013.04.004 23726147

[B13] García-Meniño I. Lumbreras P. Valledor P. Díaz-Jiménez D. Lestón L. Fernández J. Comprehensive statistical evaluation of etest. Antibiotics. (2020) 9:861. 10.3390/antibiotics9120861 . 10.3390/antibiotics9120861 PMC7761637 33287187

[B14] Goel A. Gupta V. Singhal L. Palta S. Chander J. (2021). evaluation of antibiotic synergy for carbapenem-resistant. Indian J. Med. Res. 154, 520–526. 10.4103/ijmr.IJMR_760_19 35345078 PMC9131782

[B15] Goodarzi R. Arabestani M. Alikhani M. Y. Keramat F. Asghari B. (2021). Emergence of tigecycline-resistant *Klebsiella pneumoniae* ST11 clone in patients without exposure to tigecycline. J. Infect. Dev. Ctries. 15, 1677–1684. 10.3855/jidc.15157 34898496

[B16] Gutiérrez-Gutiérrez B. Salamanca E. de Cueto M. Hsueh P. R. Viale P. Paño-Pardo J. R.. (2017). Effect of appropriate combination therapy on mortality of patients with bloodstream infections due to carbapenemase-producing Enterobacteriaceae (INCREMENT): a retrospective cohort study. Lancet Infect. Dis. 17, 726–734. 10.1016/S1473-3099(17)30228-1 28442293

[B17] Huang Y. S. Yang J. L. Wang J. T. Sheng W. H. Yang C. J. Chuang Y. C.. (2024). Evaluation of the synergistic effect of eravacycline and tigecycline against carbapenemase-producing carbapenem-resistant *Klebsiella pneumoniae*. J. Infect. Public Health 17, 929–937. 10.1016/j.jiph.2024.03.027 38599013

[B18] Korczak L. Majewski P. Iwaniuk D. Sacha P. Matulewicz M. Wieczorek P.. (2024). Molecular mechanisms of tigecycline-resistance among. Front. Cell. Infect. Microbiol. 14:1289396. 10.3389/fcimb.2024.1289396 38655285 PMC11035753

[B19] Lewis R. E. Diekema D. J. Messer S. A. Pfaller M. A. Klepser M. E. (2002). Comparison of Etest, chequerboard dilution and time-kill studies for the detection of synergy or antagonism between antifungal agents tested against *Candida* species. J. Antimicrob. Chemother. 49, 345–351. 10.1093/jac/49.2.345 11815578

[B20] Li J. Zhang H. Ning J. Sajid A. Cheng G. Yuan Z.. (2019). The nature and epidemiology of OqxAB, a multidrug efflux pump. Antimicrob. Resist. Infect. Control. 8:44. 10.1186/s13756-019-0489-3 30834112 PMC6387526

[B21] Lim T. P. Tan T. Y. Lee W. Sasikala S. Tan T. T. Hsu L. Y.. (2011). *In-vitro* activity of polymyxin B, rifampicin, tigecycline alone and in combination against carbapenem-resistant *Acinetobacter baumannii* in Singapore. PLoS ONE 6:e18485. 10.1371/journal.pone.0018485 21533030 PMC3080872

[B22] Lutgring J. D. Balbuena R. Reese N. Gilbert S. E. Ansari U. Bhatnagar A.. (2020). Antibiotic susceptibility of NDM-producing. Antimicrob Agents Chemother. (2020) 64:e00499-20. 10.1128/AAC.00499-20 32540972 PMC7449154

[B23] Ni W. Han Y. Liu J. Wei C. Zhao J. Cui J.. (2016). Tigecycline treatment for carbapenem-resistant enterobacteriaceae infections: a systematic review and meta-analysis. Medicine 95:e3126. 10.1097/MD.0000000000003126 26986165 PMC4839946

[B24] Nulsopapon P. Nasomsong W. Pongchaidecha M. Changpradub D. Juntanawiwat P. Santimaleeworagun W.. (2021). The synergistic activity and optimizing doses of tigecycline in combination with aminoglycosides against clinical carbapenem-resistant. Antibiotics. 10:736. 10.3390/antibiotics10060736 34204561 PMC8234075

[B25] Ojdana D. Gutowska A. Sacha P. Majewski P. Wieczorek P. Tryniszewska E.. (2019). Activity of ceftazidime-avibactam alone and in combination with ertapenem, fosfomycin, and tigecycline against carbapenemase-producing. Microb. Drug Resist. 25, 1357–1364. 10.1089/mdr.2018.0234 31295055

[B26] Orhan G. Bayram A. Zer Y. Balci I. (2005). Synergy tests by E test and checkerboard methods of antimicrobial combinations against Brucella melitensis. J. Clin. Microbiol. 43, 140–143. 10.1128/JCM.43.1.140-143.2005 15634962 PMC540140

[B27] Papoutsaki V. Galani I. Papadimitriou E. Karantani I. Karaiskos I. Giamarellou H.. (2020). Evaluation of *in vitro* methods for testing tigecycline combinations against carbapenemase-producing *Klebsiella pneumoniae* isolates. J. Glob. Antimicrob. Resist. 20, 98–104. 10.1016/j.jgar.2019.07.028 31398495

[B28] Papst L. Beovi,ć B. Pulcini C. Durante-Mangoni E. Rodríguez-Baño J. Kaye K. S.. (2018). Antibiotic treatment of infections caused by carbapenem-resistant Gram-negative bacilli: an international ESCMID cross-sectional survey among infectious diseases specialists practicing in large hospitals. Clin. Microbiol. Infect. 24, 1070–1076. 10.1016/j.cmi.2018.01.015 29410094

[B29] Petersen P. J. Labthavikul P. Jones C. H. Bradford P. A. (2006). *In vitro* antibacterial activities of tigecycline in combination with other antimicrobial agents determined by chequerboard and time-kill kinetic analysis. J. Antimicrob. Chemother. 57, 573–576. 10.1093/jac/dki477 16431863

[B30] Qureshi Z. A. Paterson D. L. Pakstis D. L. Adams-Haduch J. M. Sandkovsky G. Sordillo E.. (2011). Risk factors and outcome of extended-spectrum β-lactamase-producing Enterobacter cloacae bloodstream infections. Int. J. Antimicrob. Agents. 37, 26–32. 10.1016/j.ijantimicag.2010.09.009 21075605

[B31] Reese N. Lonsway D. Rasheed J. Karlsson M. (2018). 322 Correlation Between Etest and broth microdilution for colistin antimicrobial susceptibility testing of enterobacteriaceae. *Am. J. Clin. Pathol*. 149(suppl_1):S138–S9. 10.1093/ajcp/aqx126.321

[B32] Ruppé É. Woerther P. L. Barbier F. (2015). Mechanisms of antimicrobial resistance in Gram-negative bacilli. Ann. Intensive Care 5:61. 10.1186/s13613-015-0061-0 26261001 PMC4531117

[B33] Saiman L. (2007). Clinical utility of synergy testing for multidrug-resistant *Pseudomonas aeruginosa* isolated from patients with cystic fibrosis: ‘the motion for'. Paediatr. Respir. Rev. 8, 249–255. 10.1016/j.prrv.2007.04.006 17868923

[B34] Schmid A. Wolfensberger A. Nemeth J. Schreiber P. W. Sax H. Kuster S. P.. (2019). Monotherapy versus combination therapy for multidrug-resistant Gram-negative infections: systematic review and meta-analysis. Sci. Rep. 9:15290. 10.1038/s41598-019-51711-x 31664064 PMC6821042

[B35] Shi S. Xu M. Zhao Y. Feng L. Liu Q. Yao Z.. (2023). Tigecycline-rifampicin restrains resistance development in carbapenem-resistant. ACS Infect. Dis. 9, 1858–1866. 10.1021/acsinfecdis.3c00186 37669401

[B36] StataCorp (2021). Stata Statistical Software: Release 17. College Station, TX: StataCorpLLC.

[B37] Stojowska-Swedrzyńska K. Łupkowska A. Kuczyńska-Wiśnik D. Laskowska E. (2021). Antibiotic heteroresistance in *Klebsiella pneumoniae*. Int. J. Mol. Sci. 2:449. 10.3390/ijms23010449 35008891 PMC8745652

[B38] Tang H. J. Lai C. C. Chen C. C. Zhang C. C. Weng T. C. Chiu Y. H.. (2019). Colistin-sparing regimens against Klebsiella pneumoniae carbapenemase-producing *K*. pneumoniae isolates: combination of tigecycline or doxycycline and gentamicin or amikacin. J. Microbiol. Immunol. Infect. 52, 273–281. 10.1016/j.jmii.2016.03.003 27133391

[B39] The European Committee on Antimicrobial Susceptibility Testing (2024). Breakpoint Tables for Interpretation of MICs and Zone Diameters. Sweden, Version 14.0. Available at: http://www.eucast.org

[B40] Tumbarello M. Losito A. R. Giamarellou H. (2018). Optimizing therapy in carbapenem-resistant Enterobacteriaceae infections. Curr. Opin. Infect. Dis. 31, 566–577. 10.1097/QCO.0000000000000493 30379732

[B41] Wang F. Zhou Q. Yang X. Bai Y. Cui J. (2021). Evaluation of ceftazidime/avibactam alone and in combination with amikacin, colistin and tigecycline against Klebsiella pneumoniae carbapenemase-producing *K. pneumoniae* by *in vitro* time-kill experiment. PLoS ONE 16:e0258426. 10.1371/journal.pone.0258426 34648556 PMC8516195

[B42] White R. L. Burgess D. S. Manduru M. Bosso J. A. (1996). Comparison of three different in vitro methods of detecting synergy: time-kill, checkerboard, and E test. Antimicrob. Agents Chemother. 40, 1914–1918. 10.1128/AAC.40.8.1914 8843303 PMC163439

[B43] World Health Organization (2024). WHO Bacterial Priority Pathogens List, 2024: Bacterial Pathogens of Public Health Importance to Guide Research, Development and Strategies to Prevent and Control Antimicrobial Resistance. Geneva: World Health Organization.

[B44] Yaghoubi S. Zekiy A. O. Krutova M. Gholami M. Kouhsari E. Sholeh M.. (2022). Tigecycline antibacterial activity, clinical effectiveness, and mechanisms and epidemiology of resistance: narrative review. Eur. J. Clin. Microbiol. Infect. Dis. 41, 1003–1022. 10.1007/s10096-020-04121-1 33403565 PMC7785128

[B45] Zhang J. Yu L. Fu Y. Zhao Y. Wang Y. Zhao J.. (2019). Tigecycline in combination with other antibiotics against clinical isolates of carbapenem-resistant *Klebsiella pneumoniae in vitro*. Ann. Palliat. Med. 8, 622–631. 10.21037/apm.2019.09.11 31735038

[B46] Zhong X. Xu H. Chen D. Zhou H. Hu X. Cheng G.. (2014). First emergence of acrAB and oqxAB mediated tigecycline resistance in clinical isolates of Klebsiella pneumoniae pre-dating the use of tigecycline in a Chinese hospital. PLoS ONE 9:e115185. 10.1371/journal.pone.0115185 25503276 PMC4264890

