# Supplementary material for: In vitro evaluation of tigecycline synergy testing with nine antimicrobial agents against Enterobacter cloacae clinical strains
Source: Front Microbiol. 2024 Oct 18;15:1490032. doi: 10.3389/fmicb.2024.1490032 (PMC11527652; doi:10.3389/fmicb.2024.1490032)
Supplement: Supplementary file 1 [file Table_1.DOCX]

| Number of isolates | MIC of TGC alone [mg/L] | MIC of C/A alone [mg/L] | MIC of COL alone [mg/L] | MIC of ERT alone [mg/L] | MIC of GM alone [mg/L] | MIC of IMI alone [mg/L] | MIC of LEV alone [mg/L] | MIC of M/V alone [mg/L] | MIC of PMB alone [mg/L] | MIC of RIF alone [mg/L] |
| --- | --- | --- | --- | --- | --- | --- | --- | --- | --- | --- |
| 1 | R | S | R | R | R | R | R | S | R | R |
| 2 | S | S | R | R | R | R | R | R | R | R |
| 3 | R | S | R | R | R | S | R | R | R | R |
| 4 | R | S | S | R | R | R | R | R | S | R |
| 5 | R | R | S | R | R | R | R | R | S | R |
| 6 | R | S | S | R | R | R | R | R | S | R |
| 7 | R | S | S | R | R | S | R | S | R | R |
| 8 | R | R | R | R | R | S | R | S | R | R |
| 9 | R | S | R | R | R | R | R | S | R | R |
| 10 | R | S | S | S | R | S | R | S | S | R |
| 11 | R | S | S | R | R | S | R | S | S | R |
| 12 | R | S | S | R | R | S | R | S | S | R |
| 13 | R | S | S | R | R | R | R | S | R | R |
| 14 | R | S | R | R | R | S | R | R | R | R |
| 15 | R | R | S | R | R | S | R | S | R | R |
| 16 | R | R | S | R | R | S | R | S | S | R |
| 17 | R | S | S | R | R | S | R | S | S | R |
| 18 | R | S | S | R | S | S | R | S | R | R |
| 19 | R | S | S | R | R | S | R | S | R | R |
| 20 | R | S | S | R | S | R | R | R | R | R |
| 21 | R | R | S | R | R | R | R | S | S | R |
| 22 | R | S | S | R | R | S | S | S | R | R |
| 23 | R | S | R | R | R | R | R | S | R | R |
| 24 | R | S | R | R | R | S | R | S | R | R |
| 25 | R | R | S | R | S | S | R | S | S | R |
| 26 | R | S | S | R | R | S | R | S | S | R |
| 27 | R | R | R | S | R | S | R | S | R | R |
| 28 | S | S | R | S | R | S | R | S | R | R |
| 29 | S | S | R | R | R | S | R | S | R | R |
| 30 | R | S | R | S | R | R | R | S | R | R |
| 31 | R | S | R | R | R | S | R | S | R | R |
| 32 | S | S | R | R | S | S | S | S | R | R |
| 33 | R | S | R | R | R | S | R | R | R | R |
| 34 | R | R | R | R | R | S | R | S | S | R |
| 35 | S | S | R | R | R | S | R | S | S | R |
| 36 | R | S | R | R | R | S | R | S | R | R |
| 37 | S | S | R | R | R | S | R | S | R | R |
| 38 | R | S | S | R | S | S | R | S | S | R |
| 39 | R | S | R | R | R | S | R | S | S | R |
| 40 | R | S | R | R | R | R | R | S | S | R |
| 41 | R | S | R | R | R | R | R | S | S | R |
| 42 | S | S | R | R | R | S | R | S | R | R |
| 43 | R | S | R | R | R | R | R | S | R | R |
| 44 | R | S | R | R | R | R | R | S | S | R |
| 45 | R | S | R | S | S | S | S | S | R | R |
| 46 | R | S | R | S | R | S | R | S | S | R |
| 47 | R | S | R | R | R | S | R | S | R | R |
| 48 | R | S | R | R | R | S | S | S | R | R |
| 49 | R | S | R | R | R | S | S | S | S | R |
| 52 | S | S | S | R | R | R | S | S | R | R |
| 53 | S | S | R | R | S | S | S | S | S | R |
| 54 | S | S | R | R | S | S | S | S | S | R |
| 55 | S | S | S | R | S | S | S | S | S | R |
| 56 | S | S | R | R | S | S | S | S | S | R |
| 57 | S | S | R | S | S | R | R | S | S | R |
| 59 | S | S | S | S | S | R | S | S | S | R |
| 60 | S | S | R | S | S | S | S | S | S | R |
| 62 | R | S | R | S | R | S | R | S | R | R |
| 69 | R | S | R | S | R | S | R | S | S | R |
| 70 | R | S | R | S | R | S | R | S | R | R |
| 71 | R | R | R | R | R | R | R | R | S | R |
| 73 | R | S | R | R | R | R | R | S | S | R |
| 78 | R | S | R | R | R | S | R | S | R | R |
| 79 | R | S | R | R | R | S | R | S | S | R |
| 99 | R | S | R | S | R | S | R | S | S | R |
| 100 | R | S | S | R | S | S | R | S | S | R |
| 101 | R | S | R | S | S | S | S | S | R | R |
| 102 | R | S | R | S | S | S | S | S | S | R |
| 103 | R | S | R | S | S | S | S | S | S | R |
| 104 | R | S | R | R | S | R | R | S | S | R |
| 105 | R | R | R | R | R | S | R | S | S | R |
| 1_23 | R | R | S | S | R | R | R | S | R | R |
| 3_23 | R | S | R | S | R | S | R | S | R | R |
| 5_23 | R | R | R | R | R | R | R | R | R | R |
| 10_23 | R | R | S | R | R | R | R | R | S | R |
| 12_23 | R | R | R | R | S | R | R | R | S | R |
| 26_23 | R | S | S | R | R | S | R | S | S | R |
| 27_23 | R | S | R | S | R | R | R | S | R | R |
| 28_23 | R | S | R | R | R | R | R | S | R | R |
| 29_23 | R | S | R | S | R | R | S | S | S | R |

**Table 1. Phenotypes of all tested antimicrobial agents in monotherapy based on EUCAST and CLSI guidelines. Antimicrobial agents’ abbreviations: C/A – ceftazidime/avibactam; COL – colistin; ERT – ertapenem; GM – gentamicin; IMI – imipenem; LEV – levofloxacin; M/V – meropenem/vaborbactam; PB – polymyxin B; RIF – rifampicin; TGC – tigecycline. R – resistant, S – susceptible.**
