# Supplementary material for: In vitro evaluation of tigecycline synergy testing with nine antimicrobial agents against Enterobacter cloacae clinical strains
Source: Front Microbiol. 2024 Oct 18;15:1490032. doi: 10.3389/fmicb.2024.1490032 (PMC11527652; doi:10.3389/fmicb.2024.1490032)
Supplement: Supplementary file 3 [file Table_3.DOCX]

**Table 3. Phenotypes in monotherapy based on EUCAST and CLSI guidelines, and MIC values of tigecycline in combination with gentamicin, imipenem, and levofloxacin. Antimicrobial agents’ abbreviations: GM – gentamicin; IMI – imipenem; LEV – levofloxacin; TGC – tigecycline.**

| Number of isolates | Phenotype in monotherapy (GM) | TGC+GM [mg/L] | GM+TGC [mg/L] | FIC | Interpretation | Phenotype in monotherapy (IMI) | TGC+IMI [mg/L] | IMI+TGC [mg/L] | FIC | Interpretation | Phenotype in monotherapy (LEV) | TGC+LEV [mg/L] | LEV+TGC [mg/L] | FIC | Interpretation |
| --- | --- | --- | --- | --- | --- | --- | --- | --- | --- | --- | --- | --- | --- | --- | --- |
| 1 | R | 0,5 | 96 | 0,71 | **additive** | R | 0,5 | 1 | 0,50 | **synergy** | R | 0,5 | 32 | 1,33 | **indifference** |
| 2 | R | 0,064 | 8 | 0,51 | **synergy** | R | 0,19 | 1 | 0,93 | **additive** | R | 0,064 | 6 | 0,76 | **additive** |
| 3 | R | 0,38 | 96 | 0,63 | **additive** | S | 0,25 | 0,5 | 0,42 | **synergy** | R | 0,5 | 12 | 0,71 | **additive** |
| 4 | R | 1,5 | 256 | 1,25 | **indifference** | R | 0,25 | 2 | 0,50 | **synergy** | R | 0,38 | 0,25 | 0,26 | **synergy** |
| 5 | R | 1,5 | 1024 | 2,00 | **indifference** | R | 0,25 | 1 | 0,33 | **synergy** | R | 0,38 | 12 | 0,63 | **additive** |
| 6 | R | 1 | 128 | 1,50 | **indifference** | R | 0,125 | 0,5 | 0,25 | **synergy** | R | 0,5 | 16 | 1,00 | **additive** |
| 7 | R | 1 | 2 | 0,52 | **additive** | S | 0,125 | 0,125 | 0,15 | **synergy** | R | 1 | 16 | 1,00 | **additive** |
| 8 | R | 0,75 | 256 | 0,69 | **additive** | S | 0,5 | 0,25 | 0,29 | **synergy** | R | 1 | 12 | 0,63 | **additive** |
| 9 | R | 0,75 | 32 | 0,38 | **synergy** | R | 0,125 | 4 | 0,17 | **synergy** | R | 0,5 | 12 | 0,54 | **additive** |
| 10 | R | 1 | 128 | 1,50 | **indifference** | S | 0,5 | 0,064 | 0,76 | **additive** | R | 0,25 | 16 | 0,75 | **additive** |
| 11 | R | 0,5 | 48 | 1,25 | **indifference** | S | 0,75 | 0,19 | 0,94 | **additive** | R | 0,5 | 16 | 1,00 | **additive** |
| 12 | R | 0,5 | 32 | 0,58 | **additive** | S | 0,5 | 0,064 | 0,42 | **synergy** | R | 0,75 | 12 | 0,75 | **additive** |
| 13 | R | 0,5 | 1024 | 1,13 | **indifference** | R | 0,125 | 0,25 | 0,11 | **synergy** | R | 0,75 | 8 | 0,44 | **synergy** |
| 14 | R | 0,5 | 64 | 0,38 | **synergy** | S | 0,125 | 0,19 | 0,44 | **synergy** | R | 0,5 | 12 | 0,63 | **additive** |
| 15 | R | 0,5 | 4 | 0,38 | **synergy** | S | 0,125 | 0,19 | 0,21 | **synergy** | R | 0,38 | 8 | 0,50 | **synergy** |
| 16 | R | 0,5 | 96 | 0,71 | **additive** | S | 0,094 | 0,064 | 0,19 | **synergy** | R | 1 | 16 | 1,17 | **indifference** |
| 17 | R | 0,5 | 48 | 0,44 | **synergy** | S | 0,25 | 0,016 | 0,16 | **synergy** | R | 0,5 | 16 | 0,75 | **additive** |
| 18 | S | 0,5 | 0,125 | 0,33 | **synergy** | S | 0,5 | 0,125 | 0,29 | **synergy** | R | 1 | 1,5 | 0,71 | **additive** |
| 19 | R | 0,5 | 48 | 0,63 | **additive** | S | 1 | 0,047 | 0,62 | **additive** | R | 0,5 | 16 | 0,75 | **additive** |
| 20 | S | 0,125 | 0,064 | 0,29 | **synergy** | R | 0,094 | 0,016 | 0,13 | **synergy** | R | 0,064 | 0,75 | 0,21 | **synergy** |
| 21 | R | 0,5 | 64 | 0,75 | **additive** | R | 0,38 | 0,25 | 0,25 | **synergy** | R | 0,5 | 12 | 0,63 | **additive** |
| 22 | R | 0,5 | 96 | 0,75 | **additive** | S | 0,5 | 0,064 | 0,31 | **synergy** | S | 0,19 | 0,38 | 0,48 | **synergy** |
| 23 | R | 0,5 | 48 | 0,75 | **additive** | R | 0,38 | 0,19 | 0,22 | **synergy** | R | 0,75 | 6 | 0,56 | **additive** |
| 24 | R | 0,5 | 96 | 0,71 | **additive** | S | 0,5 | 0,064 | 0,46 | **synergy** | R | 0,38 | 2 | 0,32 | **synergy** |
| 25 | S | 0,75 | 0,25 | 0,63 | **additive** | S | 0,5 | 0,38 | 0,44 | **synergy** | R | 0,5 | 12 | 0,63 | **additive** |
| 26 | R | 0,38 | 24 | 0,38 | **synergy** | S | 0,75 | 0,032 | 0,44 | **synergy** | R | 0,5 | 16 | 0,75 | **additive** |
| 27 | R | 0,125 | 48 | 0,42 | **synergy** | S | 0,19 | 0,19 | 0,38 | **synergy** | R | 0,125 | 3 | 0,54 | **additive** |
| 28 | R | 0,064 | 32 | 0,67 | **additive** | S | 0,125 | 0,19 | 0,58 | **additive** | R | 0,094 | 6 | 0,75 | **additive** |
| 29 | R | 0,094 | 48 | 1,13 | **indifference** | S | 0,064 | 0,19 | 0,64 | **additive** | R | 0,064 | 3 | 0,76 | **additive** |
| 30 | R | 0,5 | 0,75 | 0,34 | **synergy** | R | 0,5 | 0,5 | 0,58 | **additive** | R | 0,5 | 12 | 0,71 | **additive** |
| 31 | R | 2 | 64 | 1,17 | **indifference** | S | 1,5 | 0,047 | 0,56 | **additive** | R | 0,75 | 12 | 0,63 | **additive** |
| 32 | S | 0,094 | 0,125 | 0,31 | **synergy** | S | 0,125 | 0,125 | 0,38 | **synergy** | S | 0,125 | 0,012 | 0,44 | **synergy** |
| 33 | R | 0,5 | 96 | 0,54 | **additive** | S | 0,75 | 0,19 | 0,38 | **synergy** | R | 0,75 | 8 | 0,50 | **synergy** |
| 34 | R | 0,75 | 48 | 0,88 | **additive** | S | 0,5 | 0,047 | 0,44 | **synergy** | R | 0,75 | 8 | 0,63 | **additive** |
| 35 | R | 0,19 | 16 | 1,17 | **indifference** | S | 0,094 | 0,19 | 0,99 | **additive** | R | 0,125 | 1,5 | 1,03 | **indifference** |
| 36 | R | 0,19 | 24 | 0,35 | **synergy** | S | 0,125 | 0,125 | 0,29 | **synergy** | R | 0,19 | 0,75 | 0,35 | **synergy** |
| 37 | R | 0,125 | 12 | 0,78 | **additive** | S | 0,064 | 0,094 | 0,58 | **additive** | R | 0,094 | 1 | 0,74 | **additive** |
| 38 | S | 0,5 | 0,19 | 0,52 | **additive** | S | 0,094 | 0,016 | 0,07 | **synergy** | R | 0,5 | 1 | 0,67 | **additive** |
| 39 | R | 0,38 | 128 | 0,51 | **additive** | S | 0,064 | 0,094 | 0,19 | **synergy** | R | 0,5 | 0,38 | 0,55 | **additive** |
| 40 | R | 0,75 | 128 | 0,88 | **additive** | R | 1 | 0,19 | 0,60 | **additive** | R | 0,5 | 16 | 0,75 | **additive** |
| 41 | R | 0,125 | 24 | 0,26 | **synergy** | R | 0,19 | 0,25 | 0,32 | **synergy** | R | 0,19 | 0,75 | 0,35 | **synergy** |
| 42 | R | 0,19 | 4 | 0,40 | **synergy** | S | 0,125 | 0,125 | 0,31 | **synergy** | R | 0,25 | 1 | 0,63 | **additive** |
| 43 | R | 0,5 | 32 | 0,63 | **additive** | R | 0,125 | 0,125 | 0,14 | **synergy** | R | 0,75 | 6 | 0,94 | **additive** |
| 44 | R | 1,5 | 3 | 0,50 | **synergy** | R | 0,75 | 0,25 | 0,27 | **synergy** | R | 1 | 32 | 1,25 | **indifference** |
| 45 | S | 0,25 | 0,125 | 0,46 | **synergy** | S | 0,19 | 0,19 | 0,63 | **additive** | S | 0,19 | 0,25 | 2,25 | **indifference** |
| 46 | R | 0,5 | 4 | 0,38 | **synergy** | S | 0,5 | 0,016 | 0,46 | **synergy** | R | 0,75 | 1 | 1,00 | **additive** |
| 47 | R | 1 | 32 | 2,00 | **indifference** | S | 0,19 | 0,094 | 0,25 | **synergy** | R | 1,5 | 2 | 2,00 | **indifference** |
| 48 | R | 0,19 | 0,75 | 0,28 | **synergy** | S | 0,125 | 0,19 | 0,25 | **synergy** | S | 0,125 | 0,094 | 0,31 | **synergy** |
| 49 | R | 0,19 | 8 | 0,25 | **synergy** | S | 0,125 | 0,094 | 0,46 | **synergy** | S | 0,25 | 0,094 | 0,41 | **synergy** |
| 52 | R | 0,25 | 12 | 0,78 | **additive** | R | 0,094 | 4 | 0,37 | **synergy** | S | 0,25 | 0,25 | 1,32 | **indifference** |
| 53 | S | 0,25 | 0,19 | 0,88 | **additive** | S | 0,094 | 0,064 | 0,36 | **synergy** | S | 0,38 | 0,006 | 0,95 | **additive** |
| 54 | S | 0,094 | 0,19 | 0,38 | **synergy** | S | 0,094 | 0,094 | 0,56 | **additive** | S | 0,19 | 0,004 | 0,51 | **additive** |
| 55 | S | 0,125 | 0,25 | 0,50 | **synergy** | S | 0,064 | 0,094 | 0,22 | **synergy** | S | 0,19 | 0,008 | 0,42 | **synergy** |
| 56 | S | 0,38 | 0,094 | 1,01 | **indifference** | S | 0,032 | 0,032 | 0,23 | **synergy** | S | 0,38 | 0,003 | 0,85 | **additive** |
| 57 | S | 0,094 | 0,125 | 0,50 | **synergy** | R | 0,016 | 6 | 0,25 | **synergy** | R | 0,016 | 0,064 | 0,07 | **synergy** |
| 59 | S | 0,094 | 0,19 | 0,31 | **synergy** | R | 0,25 | 0,5 | 0,75 | **additive** | S | 0,25 | 0,006 | 0,52 | **additive** |
| 60 | S | 0,064 | 0,125 | 0,84 | **additive** | S | 0,094 | 0,064 | 1,26 | **indifference** | S | 0,016 | 0,064 | 0,41 | **synergy** |
| 62 | R | 0,75 | 0,75 | 0,75 | **additive** | S | 0,19 | 0,064 | 0,70 | **additive** | R | 0,5 | 16 | 1,00 | **additive** |
| 69 | R | 0,75 | 0,5 | 0,50 | **synergy** | S | 0,19 | 0,25 | 0,46 | **synergy** | R | 0,38 | 12 | 0,63 | **additive** |
| 70 | R | 0,19 | 2 | 0,10 | **synergy** | S | 1 | 0,047 | 0,62 | **additive** | R | 1 | 32 | 1,50 | **indifference** |
| 71 | R | 1 | 96 | 2,00 | **indifference** | R | 0,25 | 2 | 0,31 | **synergy** | R | 0,38 | 12 | 0,76 | **additive** |
| 73 | R | 0,5 | 0,5 | 0,50 | **synergy** | R | 0,25 | 0,125 | 0,31 | **synergy** | R | 0,75 | 8 | 1,00 | **additive** |
| 78 | R | 1 | 128 | 0,83 | **additive** | S | 0,75 | 0,094 | 0,44 | **synergy** | R | 1 | 8 | 0,58 | **additive** |
| 79 | R | 0,38 | 0,38 | 0,25 | **synergy** | S | 0,25 | 0,125 | 0,25 | **synergy** | R | 1 | 16 | 1,17 | **indifference** |
| 99 | R | 1 | 32 | 1,50 | **indifference** | S | 0,25 | 0,125 | 0,25 | **synergy** | R | 0,064 | 0,19 | 0,04 | **synergy** |
| 100 | S | 1 | 1 | 1,17 | **indifference** | S | 2 | 0,094 | 0,52 | **additive** | R | 2 | 12 | 0,71 | **additive** |
| 101 | S | 0,5 | 0,5 | 0,38 | **synergy** | S | 1 | 0,012 | 0,35 | **synergy** | S | 0,5 | 0,064 | 0,38 | **synergy** |
| 102 | S | 0,38 | 0,38 | 0,57 | **synergy** | S | 1 | 0,016 | 0,56 | **additive** | S | 0,19 | 0,125 | 0,42 | **synergy** |
| 103 | S | 1,5 | 0,25 | 0,58 | **additive** | S | 0,5 | 0,125 | 0,21 | **synergy** | S | 2 | 0,5 | 1,33 | **indifference** |
| 104 | S | 0,5 | 0,25 | 0,75 | **additive** | R | 0,38 | 0,125 | 0,25 | **synergy** | R | 0,023 | 0,38 | 0,02 | **additive** |
| 105 | R | 0,75 | 96 | 0,75 | **additive** | S | 0,75 | 0,125 | 0,46 | **synergy** | R | 0,5 | 12 | 0,63 | **synergy** |
| 1_23 | R | 0,38 | 16 | 0,59 | **additive** | R | 0,38 | 0,125 | 0,32 | **synergy** | R | 0,125 | 3 | 0,18 | **synergy** |
| 3_23 | R | 0,064 | 0,5 | 0,21 | **synergy** | S | 0,19 | 0,125 | 0,21 | **synergy** | R | 0,032 | 0,25 | 0,27 | **synergy** |
| 5_23 | R | 0,25 | 2 | 0,10 | **synergy** | R | 0,25 | 0,5 | 0,33 | **synergy** | R | 0,125 | 12 | 0,42 | **synergy** |
| 10_23 | R | 0,5 | 6 | 0,52 | **synergy** | R | 0,38 | 2 | 0,51 | **additive** | R | 0,75 | 16 | 1,25 | **indifference** |
| 12_23 | S | 0,38 | 0,5 | 0,59 | **additive** | R | 0,25 | 1 | 0,25 | **synergy** | R | 0,5 | 12 | 0,71 | **additive** |
| 26_23 | R | 0,125 | 16 | 0,21 | **synergy** | S | 0,19 | 0,047 | 0,25 | **synergy** | R | 0,25 | 8 | 0,50 | **synergy** |
| 27_23 | R | 0,19 | 32 | 0,44 | **synergy** | R | 0,19 | 0,19 | 0,29 | **synergy** | R | 0,25 | 6 | 0,44 | **synergy** |
| 28_23 | R | 0,25 | 64 | 1,25 | **indifference** | R | 0,094 | 0,19 | 0,19 | **synergy** | R | 0,25 | 4 | 0,38 | **synergy** |
| 29_23 | R | 0,25 | 24 | 0,35 | **synergy** | R | 0,19 | 0,19 | 0,22 | **synergy** | S | 0,38 | 0,32 | 2,81 | **indifference** |
