# Supplementary material for: In vitro evaluation of tigecycline synergy testing with nine antimicrobial agents against Enterobacter cloacae clinical strains
Source: Front Microbiol. 2024 Oct 18;15:1490032. doi: 10.3389/fmicb.2024.1490032 (PMC11527652; doi:10.3389/fmicb.2024.1490032)
Supplement: Supplementary file 4 [file Table_4.DOCX]

**Table 4. Phenotypes in monotherapy based on EUCAST and CLSI guidelines, and MIC values of tigecycline in combination with meropenem/vaborbactam, polymyxin B and rifampicin. Antimicrobial agents’ abbreviations: M/V – meropenem/vaborbactam; PB – polymyxin B; RIF – rifampicin; TGC – tigecycline.**

| Number of isolates | Phenotype in monotherapy (M/V) | TGC+M/V [mg/L] | M/V+TGC [mg/L] | FICI | Interpretation | Phenotype in monotherapy (PMB) | TGC+PMB [mg/L] | PMB+TGC [mg/L] | FICI | Interpretation | Phenotype in monotherapy (RIF) | TGC+RIF [mg/L] | RIF+TGC [mg/L] | FICI | Interpretation |
| --- | --- | --- | --- | --- | --- | --- | --- | --- | --- | --- | --- | --- | --- | --- | --- |
| 1 | S | 0,125 | 0,016 | 0,12 | **synergy** | R | 0,19 | 0,5 | 0,29 | **synergy** | R | 0,25 | 1 | 0,33 | **synergy** |
| 2 | R | 0,094 | 0,016 | 0,38 | **synergy** | R | 0,032 | 1 | 0,38 | **synergy** | R | 0,094 | 4 | 0,71 | **addition** |
| 3 | R | 0,25 | 0,016 | 0,17 | **synergy** | R | 0,38 | 2 | 0,75 | **additive** | R | 0,125 | 2 | 0,33 | **synergy** |
| 4 | R | 0,25 | 0,016 | 0,19 | **synergy** | S | 0,25 | 0,75 | 0,54 | **additive** | R | 0,125 | 2 | 0,17 | **synergy** |
| 5 | R | 0,25 | 0,016 | 0,19 | **synergy** | S | 0,38 | 0,75 | 0,63 | **additive** | R | 0,125 | 1 | 0,15 | **synergy** |
| 6 | R | 0,125 | 0,016 | 0,14 | **synergy** | S | 0,094 | 0,25 | 0,22 | **synergy** | R | 0,125 | 1 | 0,19 | **synergy** |
| 7 | S | 0,25 | 0,016 | 0,19 | **synergy** | R | 0,38 | 1 | 0,69 | **additive** | R | 0,19 | 1 | 0,22 | **synergy** |
| 8 | S | 0,25 | 0,016 | 0,10 | **synergy** | R | 0,75 | 1 | 0,44 | **synergy** | R | 0,25 | 1 | 0,15 | **synergy** |
| 9 | S | 0,25 | 0,016 | 0,15 | **synergy** | R | 0,38 | 0,5 | 0,25 | **synergy** | R | 0,25 | 2 | 0,21 | **synergy** |
| 10 | S | 0,25 | 0,032 | 0,35 | **synergy** | S | 0,25 | 0,75 | 0,63 | **additive** | R | 0,75 | 4 | 1,08 | **indifference** |
| 11 | S | 0,5 | 0,032 | 1,00 | **additive** | S | 0,19 | 0,38 | 0,38 | **synergy** | R | 0,25 | 4 | 0,50 | **synergy** |
| 12 | S | 0,047 | 0,024 | 0,40 | **synergy** | S | 0,38 | 0,75 | 0,69 | **additive** | R | 0,125 | 3 | 0,31 | **synergy** |
| 13 | S | 0,5 | 0,016 | 0,19 | **synergy** | R | 0,5 | 0,25 | 0,25 | **synergy** | R | 0,75 | 2 | 0,31 | **synergy** |
| 14 | R | 0,75 | 0,016 | 0,40 | **synergy** | R | 0,19 | 0,5 | 0,22 | **synergy** | R | 0,75 | 2 | 0,54 | **addition** |
| 15 | S | 0,125 | 0,016 | 0,13 | **synergy** | R | 0,5 | 1 | 0,83 | **additive** | R | 1 | 2 | 0,75 | **addition** |
| 16 | S | 0,125 | 0,016 | 0,33 | **synergy** | S | 0,19 | 0,38 | 0,38 | **synergy** | R | 0,75 | 3 | 0,69 | **addition** |
| 17 | S | 0,094 | 0,016 | 0,30 | **synergy** | S | 0,5 | 1 | 0,75 | **additive** | R | 1,5 | 1,5 | 0,88 | **addition** |
| 18 | S | 0,125 | 0,016 | 0,38 | **synergy** | R | 1 | 4 | 1,67 | **indifference** | R | 0,75 | 4 | 0,42 | **synergy** |
| 19 | S | 0,25 | 0,016 | 0,47 | **synergy** | R | 0,25 | 0,38 | 0,32 | **synergy** | R | 0,75 | 2 | 0,46 | **synergy** |
| 20 | R | 0,094 | 0,032 | 0,19 | **synergy** | R | 0,19 | 0,19 | 0,35 | **synergy** | R | 0,125 | 1 | 0,21 | **synergy** |
| 21 | S | 0,19 | 0,016 | 0,22 | **synergy** | S | 0,25 | 0,38 | 0,25 | **synergy** | R | 0,5 | 3 | 0,44 | **synergy** |
| 22 | S | 0,125 | 0,016 | 0,56 | **additive** | R | 0,25 | 0,5 | 0,38 | **synergy** | R | 0,38 | 2 | 0,32 | **synergy** |
| 23 | S | 0,19 | 0,016 | 0,35 | **synergy** | R | 0,125 | 0,19 | 0,11 | **synergy** | R | 0,75 | 3 | 0,50 | **synergy** |
| 24 | S | 0,125 | 0,032 | 1,08 | **indifference** | R | 0,19 | 0,5 | 0,25 | **synergy** | R | 0,5 | 1,5 | 0,46 | **synergy** |
| 25 | S | 0,19 | 0,016 | 0,27 | **synergy** | S | 0,5 | 0,25 | 0,38 | **synergy** | R | 0,75 | 4 | 0,50 | **synergy** |
| 26 | S | 0,19 | 0,016 | 0,35 | **synergy** | S | 0,19 | 0,25 | 0,26 | **synergy** | R | 0,75 | 4 | 0,63 | **addition** |
| 27 | S | 0,125 | 0,016 | 0,29 | **synergy** | R | 0,125 | 0,5 | 0,33 | **synergy** | R | 0,75 | 3 | 1,25 | **indifference** |
| 28 | S | 0,125 | 0,016 | 1,33 | **indifference** | R | 0,047 | 0,5 | 0,25 | **synergy** | R | 0,094 | 1,5 | 0,37 | **synergy** |
| 29 | S | 0,125 | 0,016 | 1,50 | **indifference** | R | 0,064 | 0,5 | 0,38 | **synergy** | R | 0,094 | 2 | 0,63 | **addition** |
| 30 | S | 0,25 | 0,016 | 1,17 | **indifference** | R | 0,19 | 0,75 | 0,38 | **synergy** | R | 0,38 | 3 | 0,50 | **synergy** |
| 31 | S | 0,064 | 0,016 | 0,15 | **synergy** | R | 0,25 | 0,5 | 0,17 | **synergy** | R | 0,5 | 3 | 0,29 | **synergy** |
| 32 | S | 0,125 | 0,016 | 0,59 | **additive** | R | 0,19 | 0,38 | 0,51 | **additive** | R | 0,19 | 1 | 0,44 | **synergy** |
| 33 | R | 2 | 0,032 | 0,71 | **additive** | R | 0,25 | 0,38 | 0,21 | **synergy** | R | 0,75 | 2 | 0,38 | **synergy** |
| 34 | S | 1 | 0,016 | 0,67 | **additive** | S | 0,38 | 0,5 | 0,44 | **synergy** | R | 1 | 4 | 0,75 | **addition** |
| 35 | S | 0,094 | 0,016 | 1,19 | **indifference** | S | 0,094 | 0,38 | 0,68 | **additive** | R | 0,125 | 1,5 | 0,78 | **addition** |
| 36 | S | 0,125 | 0,016 | 0,34 | **synergy** | R | 0,25 | 0,75 | 0,46 | **synergy** | R | 0,094 | 2 | 0,38 | **synergy** |
| 37 | S | 0,094 | 0,023 | 0,85 | **additive** | R | 0,094 | 1,5 | 0,74 | **additive** | R | 0,094 | 1,5 | 0,68 | **addition** |
| 38 | S | 0,5 | 0,016 | 0,58 | **additive** | S | 0,5 | 0,25 | 0,46 | **synergy** | R | 0,5 | 4 | 0,46 | **synergy** |
| 39 | S | 0,25 | 0,016 | 0,75 | **additive** | S | 0,38 | 0,19 | 0,48 | **synergy** | R | 0,25 | 3 | 0,44 | **synergy** |
| 40 | S | 2 | 0,016 | 1,17 | **indifference** | S | 0,125 | 0,19 | 0,16 | **synergy** | R | 1 | 4 | 0,75 | **addition** |
| 41 | S | 0,064 | 0,016 | 0,34 | **synergy** | S | 0,25 | 0,094 | 0,43 | **synergy** | R | 0,094 | 1 | 0,21 | **synergy** |
| 42 | S | 0,125 | 0,016 | 0,42 | **synergy** | R | 0,25 | 0,5 | 0,63 | **additive** | R | 0,094 | 3 | 0,31 | **synergy** |
| 43 | S | 0,38 | 0,023 | 0,74 | **additive** | R | 0,38 | 1 | 0,88 | **additive** | R | 0,5 | 6 | 0,75 | **addition** |
| 44 | S | 0,125 | 0,016 | 0,28 | **synergy** | S | 0,19 | 0,125 | 0,11 | **synergy** | R | 2 | 4 | 0,52 | **addition** |
| 45 | S | 0,125 | 0,016 | 0,67 | **additive** | R | 0,19 | 0,75 | 0,35 | **synergy** | R | 0,19 | 2 | 0,42 | **synergy** |
| 46 | S | 1 | 0,032 | 1,17 | **indifference** | S | 0,25 | 0,75 | 0,67 | **additive** | R | 0,38 | 8 | 0,92 | **addition** |
| 47 | S | 0,25 | 0,016 | 0,75 | **additive** | R | 0,19 | 1 | 0,69 | **additive** | R | 0,38 | 4 | 0,51 | **addition** |
| 48 | S | 0,19 | 0,023 | 0,31 | **synergy** | R | 0,125 | 0,5 | 0,29 | **synergy** | R | 0,38 | 1,5 | 0,44 | **synergy** |
| 49 | S | 0,25 | 0,016 | 0,42 | **synergy** | S | 0,19 | 0,125 | 0,21 | **synergy** | R | 0,25 | 0,19 | 0,19 | **synergy** |
| 52 | S | 0,094 | 0,016 | 0,75 | **additive** | R | 0,125 | 0,5 | 0,50 | **synergy** | R | 0,19 | 1 | 0,63 | **addition** |
| 53 | S | 0,094 | 0,016 | 0,69 | **additive** | S | 0,19 | 0,25 | 0,63 | **additive** | R | 0,094 | 2 | 0,35 | **synergy** |
| 54 | S | 0,094 | 0,016 | 0,44 | **synergy** | S | 0,125 | 0,125 | 0,38 | **synergy** | R | 0,19 | 1 | 0,51 | **addition** |
| 55 | S | 0,094 | 0,016 | 0,69 | **additive** | S | 0,25 | 0,125 | 0,63 | **additive** | R | 0,125 | 1 | 0,33 | **synergy** |
| 56 | S | 0,064 | 0,016 | 0,63 | **additive** | S | 0,125 | 0,125 | 0,38 | **synergy** | R | 0,125 | 2 | 0,33 | **synergy** |
| 57 | S | 0,094 | 0,016 | 0,88 | **additive** | S | 0,25 | 0,19 | 1,19 | **indifference** | R | 0,094 | 3 | 0,56 | **addition** |
| 59 | S | 0,125 | 0,016 | 0,50 | **synergy** | S | 0,125 | 0,25 | 0,38 | **synergy** | R | 0,19 | 6 | 0,63 | **addition** |
| 60 | S | 0,032 | 0,016 | 0,76 | **additive** | S | 0,032 | 0,125 | 0,38 | **synergy** | R | 0,047 | 8 | 1,04 | **indifference** |
| 62 | S | 0,25 | 0,032 | 0,75 | **additive** | R | 0,094 | 0,75 | 0,34 | **synergy** | R | 0,38 | 8 | 0,55 | **addition** |
| 69 | S | 0,125 | 0,016 | 0,33 | **synergy** | S | 0,19 | 0,25 | 0,29 | **synergy** | R | 0,38 | 4 | 0,42 | **synergy** |
| 70 | S | 0,5 | 0,016 | 0,59 | **additive** | R | 0,125 | 0,38 | 0,25 | **synergy** | R | 0,75 | 1,5 | 0,50 | **synergy** |
| 71 | R | 0,125 | 0,19 | 0,13 | **synergy** | S | 0,094 | 0,19 | 0,19 | **synergy** | R | 0,38 | 2 | 0,51 | **addition** |
| 73 | S | 0,064 | 0,016 | 0,40 | **synergy** | S | 0,5 | 0,25 | 0,63 | **additive** | R | 0,38 | 1 | 0,46 | **synergy** |
| 78 | S | 0,5 | 0,016 | 0,51 | **additive** | R | 0,38 | 0,38 | 0,25 | **synergy** | R | 0,75 | 4 | 0,42 | **synergy** |
| 79 | S | 0,064 | 0,032 | 0,54 | **additive** | S | 0,75 | 0,75 | 0,88 | **additive** | R | 0,38 | 3 | 0,44 | **synergy** |
| 99 | S | 0,25 | 0,016 | 0,25 | **synergy** | S | 0,19 | 0,25 | 0,22 | **synergy** | R | 0,5 | 1 | 0,33 | **synergy** |
| 100 | S | 0,094 | 0,064 | 0,53 | **additive** | S | 0,25 | 0,125 | 0,10 | **synergy** | R | 1,5 | 6 | 0,50 | **synergy** |
| 101 | S | 0,125 | 0,016 | 0,37 | **synergy** | R | 0,125 | 0,75 | 0,16 | **synergy** | R | 1 | 4 | 0,42 | **synergy** |
| 102 | S | 0,125 | 0,016 | 0,31 | **synergy** | S | 0,19 | 0,5 | 0,43 | **synergy** | R | 0,5 | 2 | 0,33 | **synergy** |
| 103 | S | 1 | 0,016 | 0,67 | **additive** | S | 1,5 | 0,25 | 0,38 | **synergy** | R | 0,75 | 1,5 | 0,17 | **synergy** |
| 104 | S | 1 | 0,016 | 1,00 | **additive** | S | 0,19 | 0,125 | 0,22 | **synergy** | R | 0,5 | 3 | 0,44 | **synergy** |
| 105 | S | 0,25 | 0,064 | 0,64 | **additive** | S | 0,5 | 0,75 | 0,75 | **additive** | R | 0,75 | 3 | 0,47 | **synergy** |
| 1_23 | S | 0,19 | 0,016 | 0,47 | **synergy** | R | 0,25 | 0,75 | 0,35 | **synergy** | R | 0,38 | 4 | 0,38 | **synergy** |
| 3_23 | S | 0,19 | 0,016 | 0,47 | **synergy** | R | 0,125 | 4 | 0,09 | **synergy** | R | 0,25 | 1 | 0,33 | **synergy** |
| 5_23 | R | 0,25 | 1 | 0,58 | **additive** | R | 1 | 2 | 0,67 | **additive** | R | 1 | 4 | 0,50 | **synergy** |
| 10_23 | R | 0,032 | 0,25 | 0,09 | **synergy** | S | 0,094 | 0,38 | 0,28 | **synergy** | R | 0,5 | 6 | 0,69 | **addition** |
| 12_23 | R | 0,125 | 0,25 | 0,17 | **synergy** | S | 0,38 | 0,75 | 0,63 | **additive** | R | 0,5 | 1,5 | 0,43 | **synergy** |
| 26_23 | S | 0,5 | 0,016 | 1,00 | **additive** | S | 0,5 | 1 | 1,00 | **additive** | R | 0,19 | 2 | 0,27 | **synergy** |
| 27_23 | S | 0,125 | 0,016 | 0,63 | **additive** | R | 0,25 | 0,38 | 0,38 | **synergy** | R | 0,25 | 2 | 0,38 | **synergy** |
| 28_23 | S | 0,75 | 0,016 | 1,09 | **indifference** | R | 0,19 | 0,25 | 0,27 | **synergy** | R | 0,19 | 2 | 0,32 | **synergy** |
| 29_23 | S | 0,25 | 0,016 | 0,51 | **additive** | S | 0,25 | 0,38 | 0,42 | **synergy** | R | 0,19 | 1 | 0,19 | **synergy** |
